# Mucoepidermoid Carcinoma Superimposed on a Warthin's Tumor: A Case Report and Review of the Literature

**DOI:** 10.22038/ijorl.2020.46189.2514

**Published:** 2021-01

**Authors:** Shirin Taraz Jamshidi, Samaneh Mahjouri, Leila Vazifeh Mostaan

**Affiliations:** 1 *Department of Pathology, Faculty of Medicine, Mashhad University of Medical Sciences, Mashhad, Iran.*; 2 *Cancer Research Center, Mashhad University of Medical Sciences, Mashhad, Iran.*

**Keywords:** Mucoepidermoid carcinoma, Parotid gland, Salivary glands, Warthin's tumor

## Abstract

**Introduction:**

Warthin's tumor (WT) is the second most common neoplasm of the parotid gland and consists of two components, including lymphoid stroma and glandular epithelium. The malignant transformation of this tumor occurs most often in the lymphoid component; however, the carcinomatous transformation of the epithelial component is rare.

**Case Report:**

We present a patient who had a mass in the right mandibular angle two years before referral. A cystic mass was reported on sonography, and the patient underwent superficial parotidectomy with a pre-operative impression of lymphangiomatouse-like lesions. In the microscopic view, the sections revealed salivary gland neoplastic lesion with the diagnosis of WT. On the periphery of the neoplasm, another neoplastic lesion was observed along with infiltrative borders and diagnosis of mucoepidermoid carcinoma.

**Conclusion:**

The WT is one of the most common tumors of the salivary glands. Malignancy transformation of the WT is a rare event. However, due to the importance of the treatment type, the surgeon should consider this issue in cystic lesions suspected of WT.

## Introduction

Warthin's tumor (WT), also known as papillary cystadenoma lymphomatosum (1), is the second most common parotid neoplasm after pleomorphic adenoma that is composed of two components, including a lymphoid stroma and overlying bilayered glandular epithelium with eosinophilic cytoplasm (2,3). Multifocal and bilateral involvement that can occur simultaneously or at different times are more common in WT, compared to other salivary gland neoplasms (4). It is more common in males and its association with smoking has been emphasized. Macroscopically, it is a brown mass that is often multicystic (1). Mostly, the WT appears as a slow-growing, painless, and mobile mass (5), and ultrasonography reveals a well-defined hypoechoic mass (6). Sometimes, the diagnosis can be confirmed by needle biopsy (7).

Occasionally, the lymphoid component may become malignant; however, it is much less probable to develop carcinoma in the epithelial component. Squamous cell carcinoma (SCC) is the most common carcinoma involving the WT. Other types of carcinomas include oncocytic carcinoma, undifferentiated carcinoma, adenocarcinoma, and mucoepidermoid carcinoma (MEC) (8). It is often thought that these carcinomas originate in the epithelial component of the WT; nonetheless, it should be noted that the WT and carcinoma sometimes involve the salivary gland at the same time as collision tumors, which have not been easy to rule out. In this study, we present a patient who had MEC in the background of the WT.

## Case Report

The patient was a 36-year-old female who presented with the complaint of a right-sided facial mass. She had noticed this painless mass two years before referral. On examination, there was a soft, round, and mobile mass in the right mandibular angle. The overlying skin was erythematous and adhered to the mass. Facial nerve function was normal, no lymphadenopathy was found, and a cystic lesion was reported on ultrasonography. Following that, the patient underwent mass resection surgery with a pre-operative diagnosis of lymphangioma and WT. Inflammation and infection in the cystic component cause the lesion to adhere to the skin. Therefore, adhesion did not necessarily mean a malignant lesion. In addition, in the classical approach, parotidectomy can be performed even in malignant lesions. Finally, due to financial problems, the patient refused to have a CT scan or fine needle biopsy. After general anesthesia, superficial parotidectomy was performed with the preservation of the facial nerve, and the lymph nodes of zone II were removed. The specimen was submitted for histopathological evaluation.

**Fig 1:**
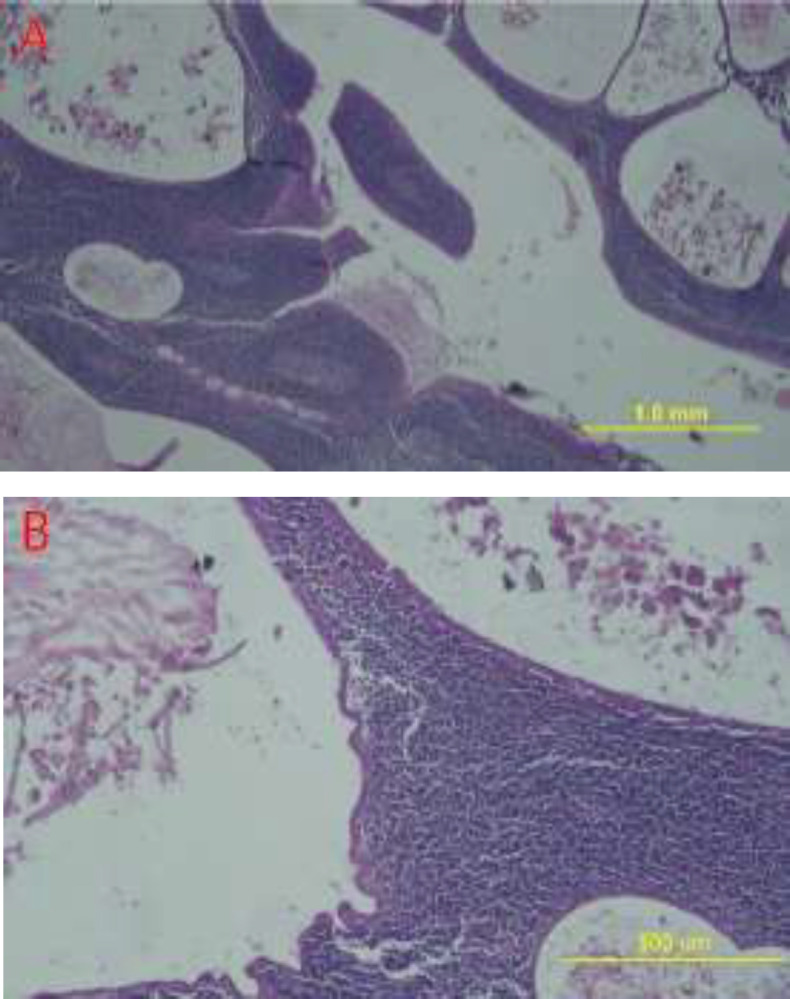
Hematoxylin and eosin staining of the Warthin's Tumor consisting of lymphoid and epithelial components (Olympus Microscope CX31)

On gross examination, the excised tissue included the superficial lobe of the parotid gland and overlying skin measuring 6×5.5×5 cm. The cut surface showed a 1.5-cm soft gray-white area with partial circumscription and cystic spaces. In the microscopic view, the sections revealed salivary gland tissue with neoplastic lesions consisting of cystic structures covered by two layers of cells with nuclei of no significant atypia and eosinophilic cytoplasm. The underlying stroma contained lymphomononuclear cells with lymphoid follicle formation (Fig. 1).

**A:** Low magnification showing the multicystic structure. **B:** A dense lymphoid stroma is observed beneath the double-layered epithelium.

**Fig 2:**
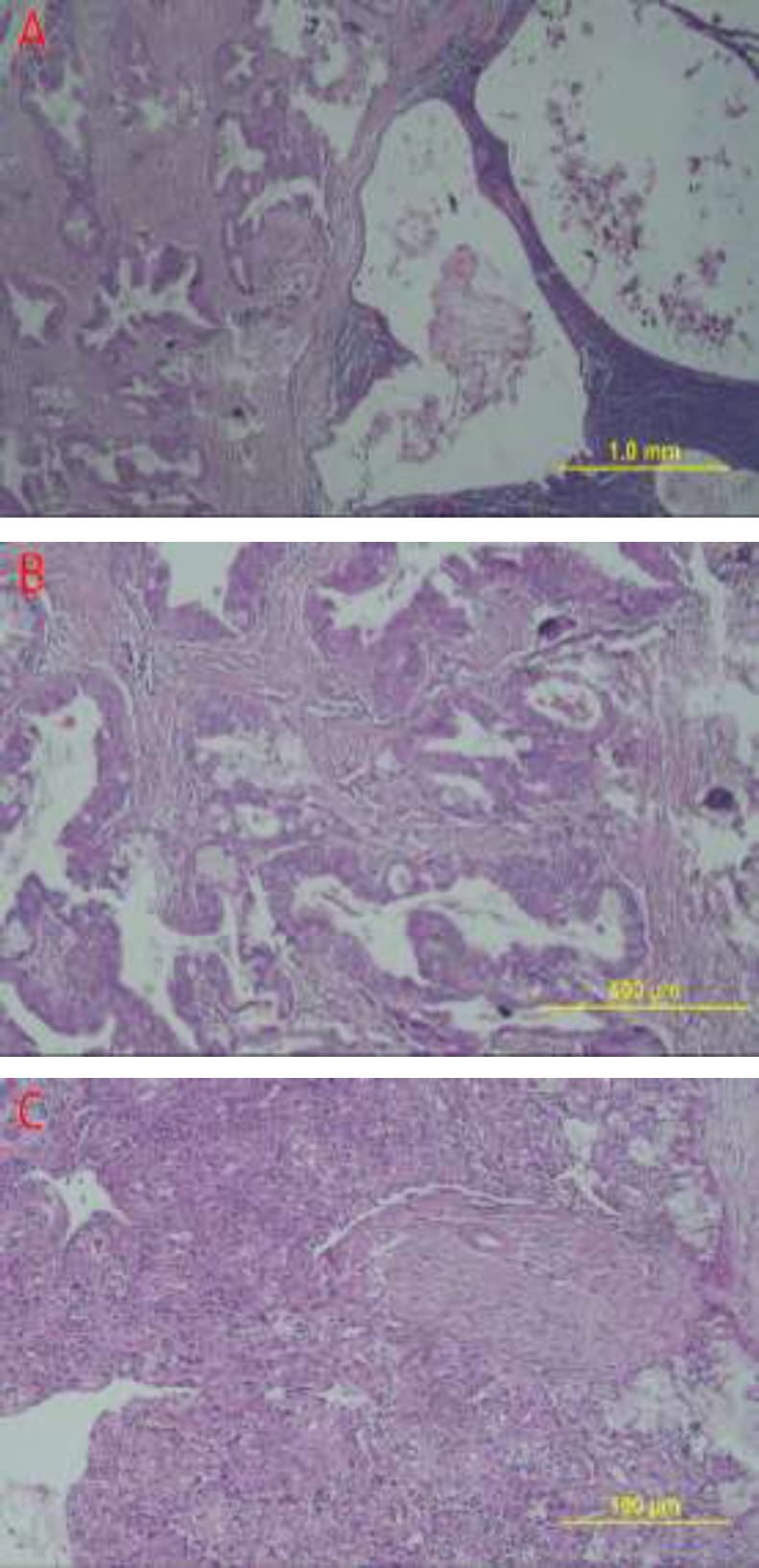
Hematoxylin and eosin-stained sections showing the MEC arising in the background of the Warthin's Tumor (Olympus Microscope CX31).

On the periphery of the neoplasm, another neoplastic lesion was observed with infiltrative borders composed of mucous-secreting glands with extensive cystic areas and few solid sheets of squamoid and intermediate cells with clear cytoplasms, mild to moderate nuclear atypia, low mitotic rate, perineural invasion, and no necrosis (Fig. 2).

**A:** Low magnification showing invasive glandular component. **B:** Higher magnification reveals mucus-secreting glands. **C:** Solid sheets of squamoid and intermediate cells with clear cytoplasms. Perineural invasion is noted on the right side.

Accordingly, the diagnosis of MEC in the background of WT was established. Dermal and deep margin involvement was observed in some foci, and the lymph nodes showed reactive features. After surgery, the patient underwent radiation therapy, and in the 5-year follow-up, the patient was in good general condition and reported no problems with the physical examination; however, she refused to take further diagnostic procedures.

## Discussion

The WT often occurs in the parotid gland and adjacent lymph nodes; however, it sometimes occurs in the minor salivary glands (1). In its classical form, it appears as a painless and slow-growing mass. Infection or malignancy should be suspected if the growth of the tumor suddenly accelerates (7). The cause of malignant transformation in the epithelial part of the WT tumor has not been fully understood. Ischemia, hypoxia, or chronic local inflammation may play an important role in transforming epithelial metaplasia into atypical hyperplasia (9). Malignant transformation is often observed in lymphoid cells. Carcinoma is very rare (0.3%) in the epithelial component (8), and the following cases have been reported based on our reviews (Table.1).

The WT mainly occurs in the elderly (7). In the reported cases of MEC involving WT, the youngest and oldest patients were a 9-year-old female and two 73-year-old female and male, respectively. It should also be considered that sometimes MEC coincides with WT in the salivary gland. These cases are called ''Collison tumors'' (10). The presence of a transitional zone between the epithelial components of WT and MEC may help confirm the diagnosis (7).

It can also be difficult to differentiate between goblet cell metaplasia in the WT from mucous cells of a low-grade MEC (19). Diagnostic aid, in this case, is the infiltration of lymphoid tissue and capsule with MEC. The WT and MEC have two types of epithelial cells. The MEC is composed of epidermoid and mucous cells while WT is composed of basal and oncocytic cells. The WT basal cells and MEC epidermoid cells are both immunoreactive for CK5/6, CK 34βe12, and p63; moreover, the mucous and oncocytic cells are both positive for CK7 (7). In the present case, the components of the WT and MEC tumors were distinct, and there was no need for immunohistochemistry to determine their nature.

**Table 1:** Summary of 21 Cases of Mucoepidermoid Carcinoma Involving Warthin's Tumor

**Clinical Follow-Up**	**MEC** **Grade**	**MEC** **Size (mm)**	**WT** **Size (mm)**	**Side**	**Gender**	**Age (y)**	**Reference**
NORM, 72 months	NA	NA	18	Left	M	60	Gadient and Kalfayan, 1975 (10)
NORM, time of follow-up not Specified	II	NA	40	Bilateral	M	73	Seifert, 1997 (11)
NA	NA	NA	20	Left	F	66	Saku et al, 1997 (12)
NORM, 30 months	NA	NA	28	Left	M	58	Nagao et al,1998 (13)
NORM, 34 months	NA	NA	45	Right	F	54	
Lost to follow-up	II	17	20	Right	F	45	Williamson et al, 2000 (14)
NORM, 52 months	II	7	17	Right	M	40	
Lost to follow-up	I	3	32	NA	M	39	
NORM, 30 months	I	15	40	Right	M	60	
NORM, 8 months	I	15	60	Left	F	70	
NA	NA	NA	NA	NA	F	35	Mardi et al, 2007 (15)
NA	NA	NA	NA	Right	F	55	Mohapatra et al, 2012 (9)
24 months, NORM	NA	NA	18	Left	M	61	Smotka et al, 2015 (16)
NORM until death (36 months)	II	NA	47	Left	F	73	Hakeem et al, 2016 (17)
25 months, NORM	NA	NA	36	Right	F	43	Yu et al, 2016 (7)
48 months, NORM	NA	NA	42	Right	M	40	
63 months, NORM	NA	NA	20	Left	M	63	
69 months, NORM	NA	NA	25	Right	F	26	
25 months, NORM	NA	NA	5- 25	Right	M	56	
NA	NA	NA	NA	Left	F	9	Citak et al, 2019 (18)
60 months, NORM	NA	NA	15	Right	F	36	Current case


The MECs arising in the background of the WT should be differentiated from other similar lesions. Sometimes, the squamous and mucous metaplasia occur in WT; however, the squamous metaplasia occurs in almost 7.5% of the WT. The key difference between them is the absence of atypical cells and infiltrative growth in metaplasia (11). In the present case, mucin produced an intermediate and squamous component with an invasive margin that caused adhesion to the dermis; moreover, a perineural invasion was noted; however, such a view was not observed in metaplastic WT.

Another issue to note is the MEC's differentiation from the SCC in the WT context. In this case, the presence of mucous cells that are positive for CEA and Alcian Blue/PAS staining is in favor of MEC (8). The third point to note is that WT sometimes acts as a receptor for a metastatic tumor. Cases of renal and lung neoplasms metastasized to the WT have been reported (7).

 In these cases, the diagnosis is made based on clinical and radiological signs. 

In microscopic evaluation, the transitional zone is not observed in metastatic lesions. It is important to differentiate the metastasis from the primary tumor due to different management strategies. In our case, other organs were also examined and the metastatic lesion was excluded. Treatment in patients with MEC superimposed on the WT is mainly surgical resection with lymph node dissection. These patients are susceptible to recurrence and metastasis; therefore, the patient should be followed-up for a long time. No recurrence or metastasis was observed in the 5-year follow-up of our patient. 

## Conclusion

The MEC in the background of WT is a rare finding, and the tumor can mimic a benign lesion clinically as in our case. Accordingly, correct preoperative diagnosis is important from a therapeutic point of view. 

## Ethical Approval

This study was approved by the Ethics Committee of Mashhad University of Medical Sciences, Mashhad, Iran.
